# Ethnic differences in physical and mental multimorbidity in working age adults with a history of depression and/or anxiety

**DOI:** 10.1017/S0033291722003488

**Published:** 2023-10

**Authors:** Amy Ronaldson, Jorge Arias de la Torre, Matthew Broadbent, Mark Ashworth, David Armstrong, Ioannis Bakolis, Stephani L. Hatch, Matthew Hotopf, Alex Dregan

**Affiliations:** 1 Institute of Psychiatry, Psychology and Neuroscience (IoPPN), King's College London, London, UK; 2 CIBER Epidemiology and Public Health (CIBERESP), Madrid, Spain; 3 Institute of Biomedicine (IBIOMED), University of Leon, Leon, Spain; 4 NIHR Maudsley Biomedical Research Centre, King's College London, London, UK; 5 School of Population Health & Environmental Sciences, King's College London, London, UK; 6 ESRC Centre for Society and Mental Health, King's College London, London, UK; 7 South London and Maudsley NHS Foundation Trust, London, UK

**Keywords:** Anxiety, cluster analysis, common mental health disorders, depression, ethnicity, multimorbidity

## Abstract

**Background:**

The current study used data from an ethnically diverse population from South London to examine ethnic differences in physical and mental multimorbidity among working age (18–64 years) adults in the context of depression and anxiety.

**Method:**

The study included 44 506 patients who had previously attended Improving Access to Psychological Therapies services in the London Borough of Lambeth. Multinomial logistic regression examined cross-sectional associations between ethnicity with physical and mental multimorbidity. Patterns of multimorbidity were identified using hierarchical cluster analysis.

**Results:**

Within 44 056 working age adults with a history of depression or anxiety from South London there were notable ethnic differences in physical multimorbidity. Adults of Black Caribbean ethnicity were more likely to have physical multimorbidity [adjusted relative risk ratio (aRRR) = 1.25, 95% confidence interval (CI) 1.15–1.36] compared to adults of White ethnicity. Relative to adults of White ethnicity, adults of Asian ethnicity were more likely to have physical multimorbidity at higher thresholds only (e.g. 4 + conditions; aRRR = 1.53, 95% CI 1.17–2.00). Three physical (atopic, cardiometabolic, mixed) and three mental (alcohol/substance use, common/severe mental illnesses, personality disorder) multimorbidity clusters emerged. Ethnic minority groups with multimorbidity had a higher probability of belonging to the cardiometabolic cluster.

**Conclusion:**

In an ethnically diverse population with a history of common mental health disorders, we found substantial between- and within-ethnicity variation in rates of physical, but not mental, multimorbidity. The findings emphasised the value of more granular definitions of ethnicity when examining the burden of physical and mental multimorbidity.

## Introduction

Multimorbidity (two or more co-existing health conditions) is increasingly common affecting approximately 27% of United Kingdom (UK) adults in primary care (Cassell et al., 2018). Multimorbidity poses a considerable challenge to patients and their caregivers, clinicians, and health and care systems seeing as it is associated with poor health-related quality of life (Makovski, Schmitz, Zeegers, Stranges, & van den Akker, 2019), functional decline (Storeng, Vinjerui, Sund, & Krokstad, 2020), increased healthcare utilisation (Glynn et al., 2011), and mortality (Willadsen et al., 2018).

There are known sociodemographic disparities in the development of multimorbidity. Rates of multimorbidity are known to be higher among women and people living in socially deprived areas (Ingram et al., 2021; Marengoni et al., 2011). There are also ethnic inequalities in multimorbidity with higher burden and faster accumulation of long-term conditions seen among ethnic minority groups, even after adjusting for socioeconomic status (Verest et al., 2019). Studies from the US have shown that Black people have higher levels of multimorbidity than White people (Johnson-Lawrence, Zajacova, & Sneed, 2017; Quiñones, Liang, Bennett, Xu, & Ye, 2011; 2019; Rocca et al., 2014; St Sauver et al., 2015) including more complex multisystem multimorbidity (i.e. multimorbidity that affects multiple organ systems) (Kalgotra, Sharda, & Croff, 2020; Quiñones et al., 2021).

Less is known about associations between ethnicity and multimorbidity in the UK, particularly at local community levels where the makeup of ethnic minority groups differs from that observed at national UK levels. For example, while South East Asians are the largest ethnic minority group in the UK (Office of National Statistics, 2020), within South London people of Black ethnicity are the predominant ethnic minority group. The evidence about differences in patterns of multimorbidity between people from Black ethnic minority groups relative to those from a White background are less available within a UK context. Even less available are data about potential within-ethnic group differences (e.g. Black Caribbean *v.* Black African) in the patterning of mental health related multimorbidity. Such inequalities are probably exacerbated in areas of high social deprivation, like South London (e.g. Lambeth, Lewisham, Southwark and Croydon boroughs). A recent study using data from South London found higher rates of multimorbidity among Black and South Asian ethnic groups compared to those of White ethnicity (Ashworth et al., 2019). However, this study focused on a highly restricted definition of multimorbidity that included a limited number of conditions associated with high healthcare and social care demands. Moreover, most of the existing evidence to date concentrates on specific age groups – commonly older adults – and less evidence is available among working age populations (i.e. the age range at which people are typically engaged in paid or unpaid work – 18–64 years in the UK).

It is estimated that around 40% of people with depression and/or anxiety also have a long-term physical condition (Naylor et al., 2012). While the evidence suggests that physical multimorbidity is higher in some ethnic minority groups, the same ethnic differences are not being seen in rates of depression and anxiety (Rees et al., 2016). Consequently, ethnic differences in physical multimorbidity in the context of depression and anxiety may differ to those seen when more general definitions of multimorbidity are used. In England, Improving Access to Psychological Therapies (IAPT) services provide evidence-based therapy to people with mild to moderate depression and anxiety disorders within the English National Health Service (NHS). IAPT services record detailed demographic information pertaining to ethnicity, and routinely monitor patients' mental and physical wellbeing over time. Thus IAPT data provide a rich and unique context in which to examine ethnic differences in physical multimorbidity amongst people with depression and/or anxiety, particularly in light of the recent introduction of specific referral criteria encouraging IAPT referrals for people with long-term physical health conditions (NHS England, 2018).

The current study used linked IAPT and primary care data in an ethnically diverse population from South London to examine ethnic differences in physical and mental multimorbidity among working age adults (18–64 years), in the context of depression and anxiety. The key hypothesis was that working age adults from ethnic minority backgrounds would have higher rates of physical and mental multimorbidity compared to working age adults from a White ethnic background. Given documented ethnic differences in the type of conditions that co-occur in patients with multimorbidity (Kalgotra et al., 2020; Quiñones et al., 2021), we used hierarchical cluster analysis to identify clusters of physical and mental conditions and examined ethnic differences in cluster membership amongst those with multimorbidity. This data driven approach allowed us to capture the complexity of multimorbidity more than using simple disease counts or clinical classifications (e.g. ICD-10 categories) (Majnarić, Babič, O'Sullivan, & Holzinger, 2021). We hypothesised variability in the multimorbidity cluster membership between ethnic minority adults and their White ethnic group counterparts.

## Methods

### Sample and study design

This was a cross-sectional observational study using linked data from electronic health records from the IAPT services in the London Borough of Lambeth and Lambeth DataNet (LDN) (extracted on 22nd March 2021) (Perera et al., 2016). Lambeth is one of the most densely populated areas in the UK with a younger age profile (i.e. 44% people aged 20–39 years) compared to other London boroughs and the rest of the country (Lambeth Council, 2016). Lambeth is an ethnically diverse borough with 60% of the population describing their ethnicity as other than White British (Lambeth Council, 2017). It is also one of the most deprived areas in England with 31% of people in the borough living in areas of high deprivation (Lambeth Council, 2017).

IAPT services were established in England to provide evidence-based psychological therapies to people with mild to moderate depression and anxiety disorders within the NHS (Clark, 2011). LDN is an anonymised dataset consisting of Read-coded clinical data from general practices in the London Borough of Lambeth which was set up as a local resource to allow for the assessment of local health inequalities, particularly related to ethnicity and social deprivation (Healthwatch Lambeth, 2017). Primary care records from LDN were linked with IAPT data. Therefore, the primary sample consisted of 47 097 adults who were registered with a general practitioner (GP) in the London Borough of Lambeth and who had accessed Lambeth IAPT services before March 2021. We envisaged that ethnicity might influence physical and mental multimorbidity both independently as well as interdependently, whereby early inequalities in mental disorders for ethnic minority groups might accelerate future risk of physical multimorbidity. Assessing associations amongst people who had accessed IAPT services for depression/anxiety allowed us to account for this. Ethical approval was granted by the Oxford Research Ethics Committee (18/SC/0372).

### Ethnicity

Detailed information about ethnicity was gathered in IAPT services through self-report. In line with another study carried out in Lambeth IAPT users (Harwood et al., 2021), we included seven ethnic groups: White (White British and White Other), Black Caribbean, Black African, Black Other, Asian (e.g. Bangladeshi, Indian, Pakistani), Mixed ethnicity, and Other (e.g. Arab/Middle Eastern, Other Latin American). A detailed description of how the ethnic groups were derived is provided in online Supplementary Table S1. We felt it was important to explore black ethnicities separately due to their distinct experiences and disaggregated the Black ethnic group into three categories as we had a sufficient sample size to do this. Unfortunately, the Asian group was not large enough to allow us to explore Asian ethnic groups separately.

### Physical and mental multimorbidity status

Physical and mental multimorbidity status were measured separately and were derived from counts of health conditions recorded in primary care records (LDN) up to the date of data extraction (March 2021). An adapted list of conditions were classified based on previous work on multimorbidity (Barnett et al., 2012) – a total of 32 physical (e.g. cancer, diabetes) and 10 mental (e.g. schizophrenia, bipolar disorder) conditions were included (see online Supplementary Table S2 for a detailed list of conditions). In the current study, physical and mental multimorbidity status measures were defined using a cut-off of two or more conditions for each study participant (Johnston, Crilly, Black, Prescott, & Mercer, 2019). We created multi-categorical physical and mental multimorbidity variables that grouped participants into; 0 or 1 condition (no multimorbidity); 2 conditions; 3 conditions; 4 or more conditions.

### Physical and mental multimorbidity clusters

We used agglomerative hierarchical cluster analysis in those with physical and mental multimorbidity to identify distinct subgroups of participants with similar physical and mental disease combinations respectively. We first performed an average linkage cluster analysis based on Jaccard's similarity coefficient and then performed k-means cluster analysis using the Calinski-Harabasz index (elbow method) to identify the optimal number of clusters (Collerton et al., 2016). To characterise each cluster, we compared the prevalence of each condition within a specific cluster to its prevalence in the total sample with multimorbidity. We assigned a condition to a cluster when it had ‘higher than average prevalence’, i.e., when the prevalence in the cluster was ⩾20% higher than the prevalence in the total sample (i.e. the observed/expected (O/E) ratio was 1.2:1 or higher) (Collerton et al., 2016).

### Covariates

Covariates included age (Triolo et al., 2020), gender (male, female, other) (Agur, McLean, Hunt, Guthrie, & Mercer, 2016), neighbourhood deprivation (Pathirana & Jackson, 2018), body mass index (BMI) (Romain, Marleau, & Baillot, 2019), smoking status (Dhalwani et al., 2017), and total number of IAPT episodes of care. These variables were directly obtained or derived from either IAPT services or LDN data. Age was the patient age when data were extracted (March 2021). Gender was gathered through self-report in IAPT services. Neighbourhood deprivation was measured using the Index of Multiple Deprivation (IMD) (Noble et al., 2007) 2015 classification at lower super output area recorded in LDN. IMD scores ranged from one to 10 with lower deciles indicating higher neighbourhood deprivation. BMI and smoking status were obtained from GP records via LDN. The latest recorded BMI value and the latest recorded smoking status (never smoked, ex-smoker, current smoker) were included in fully adjusted models (i.e. models adjusted for all covariates). We also adjusted for total number of IAPT episodes of care (i.e. the number of times a person received IAPT treatment before March 2021) which acted as a proxy for depression/anxiety severity seeing as increased symptoms of depression/anxiety are associated with multimorbidity (Triolo et al., 2020). Patient Health Questionnaire (PHQ)-9 (Kroenke, Spitzer, & Williams, 2001) and Generalised Anxiety Disorder Assessment (GAD)-7 (Spitzer, Kroenke, Williams, & Löwe, 2006) scores from the baseline assessment at each patients' first episode of care in IAPT services were included in descriptive statistics in order to provide further insight into depression and anxiety severity respectively. They were not included as covariates as we believe the number of IAPT episodes of care provides a better indicator of the severity and longevity of depression/anxiety more than pre-treatment PHQ-9/GAD-7 scores which were potentially measured some years ago.

### Statistical analysis

Variables were summarised as medians and interquartile ranges (IQR) and frequencies. Comparisons between ethnic groups were performed using Kruskall–Wallis and chi squared tests. We also investigated whether there were differences between the analytical sample and those excluded from the study due to missing data. Cross-sectional associations between ethnicity and both physical and mental multimorbidity status were measured using multinominal logistic regression. Adjusted relative risk ratios (aRRR) together with their respective 95% confidence intervals (CI) were obtained. All models were fitted with increasing degrees of covariate adjustment: unadjusted, age- and sex-adjusted, and fully adjusted (all covariates). In fully adjusted models, we controlled for *a priori* confounders including age, gender, neighbourhood deprivation, BMI, smoking, and total number of IAPT episodes. To ensure the most parsimonious model, we tested the association between ethnicity and all study covariates. Covariates that were statistically significant were retained for further analysis. To adjust for multiple comparisons we applied a Bonferroni correction to these analyses leading to an alpha level of 0.003.

Adjusted multinomial logistic regression was employed to examine ethnic differences in the likelihood of physical and mental multimorbidity cluster membership, with the largest cluster acting as the reference group. aRRR together with their respective 95% CIs were obtained. In fully adjusted models, we controlled for *a priori* confounders including age, gender, neighbourhood deprivation, BMI, smoking, and total number of IAPT episodes.

Data were missing for several variables: neighbourhood deprivation (2.8%), BMI (11.3%), and smoking status (2.0%). Multiple imputation using chained equations with 10 imputations was performed to deal with missing data (Azur, Stuart, Frangakis, & Leaf, 2011). All fully adjusted analyses were based on imputed data.

All analyses were conducted in STATA 15.1 (Stata Corp LLP, College Station, TX).

## Results

### Sample characteristics

As the focus of the study was on people of working age (18–64 years), after removing those over 64 years (*N* = 1222) and those with missing data on ethnicity (*N* = 1369), the final sample comprised 44 506 working age adults who had accessed IAPT services in Lambeth. Sample characteristics are described in Table 1. The median age of the overall sample was 37.4 years (IQR = 31.3 to 46.6) and 65% were female. Compared to those in the White ethnic group, working age adults from ethnic minority groups were more likely to be female and were more likely to live in a deprived neighbourhood. Rates of overweight and obesity were higher among all ethnic minority groups compared to the White ethnic group, while working age adults of Black Caribbean, Black Other, and Mixed ethnicity were more likely to be current smokers than those of White ethnicity. In terms of access to IAPT services, on average, ethnic minority groups had more IAPT episodes of care than those in the White ethnic group.

**Table 1.** Sample characteristics of the overall sample and by ethnic group. Figures are numbers and frequencies, unless otherwise specified

	Overall (*n* = 44 506)	White (*n* = 29564, 66%)	Black Caribbean (*n* = 4816, 11%)	Black African (*n* = 2010, 5%)	Black Other (*n* = 1109, 3%)	Asian (*n* = 2114, 5%)	Mixed (*n* = 3165,7%)	Other (*n* = 1728, 4%)	*p* value
Age [Median, (IQR)]	37.4 (31.3–46.6)	36.8 (31.3–45.3)	40.7 (32.4–52.6)	37.1 (29.1–48.1)	41.9 (33.3–52.9)	38.1 (31.9–46.6)	35.1 (29.1–43.1)	40.1 (32.8–49.2)	<0.001
Female	29 054 (65)	18 852 (63)	3346 (69)	1379 (68)	774 (70)	1350 (64)	2231 (71)	1122 (65)	<0.001
IMD decile [Median (IQR)]	3 (2–5)	4 (3–5)	3 (2–4)	3 (2–4)	3 (2–4)	4 (2–5)	3 (2–4)	3 (2–4)	<0.001
BMI									<0.001
Obese	6788 (15)	6463 (12)	1372 (29)	524 (26)	319 (29)	320 (15)	525 (17)	265 (15)	
Overweight	12 315 (28)	7639 (26)	1580 (33)	662 (33)	382 (34)	617 (29)	859 (27)	576 (33)	
Normal weight	23 846 (54)	17 387 (59)	1745 (36)	771 (38)	384 (35)	1075 (51)	1652 (52)	832 (48)	
Underweight	1557 (4)	1075 (4)	119 (3)	53 (3)	24 (2)	102 (5)	129 (4)	55 (3)	
Smoking status									<0.001
Current	14 406 (32)	9486 (32)	1779 (37)	505 (25)	360 (33)	572 (27)	1169 (37)	535 (31)	
Ex-smoker	7947 (18)	5750 (19)	762 (16)	224 (11)	165 (15)	253 (12)	562 (18)	231 (13)	
Never smoked	22 153(50)	14 328 (49)	2275 (47)	1281 (64)	584 (53)	1289 (61)	1434 (45)	962 (56)	
IAPT episodes (Median (IQR))	1 (1–1)	1 (1–1)	1 (1–2)	1 (1–1)	1 (1–2)	1 (1–1)	1 (1–2)	1 (1–2)	<0.001
PHQ-9^a^ (Median (IQR))	15 (10–19)	14 (9–19)	17 (12–21)	16 (11–21)	17 (12–21)	17 (12–21)	16 (11–20)	16 (11–21)	<0.001
GAD-7^a^ (Median (IQR))	14 (10–17)	13 (9–17)	15 (10–18)	14 (10–18)	15 (10–18)	15 (11–18)	14 (10–18)	15 (10–18)	<0.001
Physical MM	9923 (22)	6078 (21)	1473 (31)	421 (21)	354 (32)	501 (24)	747 (24)	349 (20)	<0.001
0/1 condition	34 583 (78)	23 486 (79)	3343 (69)	1589 (79)	755 (68)	1613 (76)	2418 (76)	1379 (80)	
2 conditions	6592 (15)	4171 (14)	908 (19)	264 (13)	207 (19)	288 (14)	538 (17)	216 (13)	
3 conditions	2162 (5)	1253 (4)	356 (7)	98 (5)	83 (7)	142 (7)	150 (5)	80 (5)	
4 + conditions	1169 (3)	654 (2)	209 (4)	59 (3)	64 (6)	71 (3)	59 (2)	53 (3)	
Mental MM	1682 (4)	1113 (4)	207 (4)	54 (3)	48 (4)	60 (3)	149 (5)	51 (3)	0.002
0/1 condition	42 824 (96)	28 451 (96)	4609 (96)	1956 (97)	1061 (96)	2054 (97)	3016 (95)	1677 (97)	
2 conditions	1293 (3)	852 (3)	158 (3)	45 (2)	33 (3)	51 (2)	114 (4)	40 (3)	
3 conditions	300 (1)	195 (1)	35 (1)	8 (0)	14 (1)	9 (0)	29 (1)	10 (1)	
4 + conditions	89 (0)	66 (0)	14 (0)	1 (0)	1 (0)	–	6 (0)	1 (0)	

BMI, body mass index; GAD-7, generalised anxiety disorder assessment; IAPT, Improving Access to Psychological Therapies; IMD, Index of Multiple Deprivation; PHQ-9, Patient Health Questionnaire-9.

a Baseline PHQ-9 and GAD-7 scores are taken from the first episode of care in IAPT services.

Rates of physical multimorbidity significantly differed across ethnic groups with higher rates seen across most ethnic minority groups relative to the White ethnic group. Although there were significant ethnic differences in rates of mental multimorbidity these differences were more mixed with higher rates seen in Black Caribbean, Black Other, and Mixed ethnic groups and lower rates seen in Black African, Asian, and Other ethnic groups when compared to the White ethnic group.

We investigated differences between the analytical sample (*N* = 44 056) and those excluded from the study on the basis of age and missing ethnicity data (*N* = 2591) (online Supplementary Table S3 in Supplementary Material). Excluded participants differed from the analytical sample on many sociodemographic factors, had higher PHQ-9 and GAD-7 scores at baseline, and had higher levels of physical and mental multimorbidity. We also examined ethnic differences in multimorbidity in those with complete data on all variables (*N* = 38 292) in order to assess whether imputing data affected the results of the study. Results were very similar (online Supplementary Table S4 in Supplementary Material).

### Ethnic differences in the prevalence of individual long-term conditions

The five most prevalent long-term conditions amongst those with physical and mental multimorbidity for each ethnic group are displayed in Table 2. In adults with physical multimorbidity, psoriasis/eczema emerged as the most prevalent long-term physical condition across all ethnic groups. Diabetes was among the top five most common physical conditions within all ethnic groups (apart from the White and Mixed groups), with the highest rate seen in adults of Black African ethnicity (26%). Psychosis was the most prevalent mental condition for working age adults of Black African ethnicity (43%), while substance misuse emerged as the most prevalent condition for adults of Black Caribbean ethnicity (47%).

**Table 2.** Ethnic differences in the five most prevalent long-term conditions amongst adults with physical or mental multimorbidity (2 or more conditions)

Physical long-term conditions								
	Overall (*n* = 9923)	White (*n* = 6078, 61%)	Black Caribbean (*n* = 1473, 15%)	Black African (*n* = 421, 4%)	Black Other (*n* = 354, 4%)	Asian (*n* = 501, 5%)	Mixed (*n* = 747, 8%)	Other (*n* = 349, 4%)
1	Psoriasis/eczema 61%	Psoriasis/eczema 61%	Psoriasis/eczema 63%	Psoriasis/eczema 52%	Psoriasis/eczema 63%	Psoriasis/eczema 65%	Psoriasis/eczema 68%	Psoriasis/eczema 54%
2	Asthma 46%	Asthma 46%	Asthma 45%	Hypertension 38%	Asthma 45%	Asthma 45%	Asthma 53%	Asthma 35%
3	IBS 26%	IBS 29%	Hypertension 35%	Asthma 36%	Hypertension 33%	IBS 26%	IBS 24%	IBS 25%
4	Hypertension 19%	Hearing 17%	Diabetes 21%	Diabetes 26%	Diabetes 23%	Diabetes 24%	Hearing 16%	Hypertension 23%
5	Hearing 16%	Hypertension 14%	IBS 19%	IBS 14%	IBS 20%	Hypertension 21%	Hypertension 15%	Diabetes 18%
Mental long-term conditions								
	Overall (*n* = 1682)	White (*n* = 1113, 66%)	Black Caribbean (*n* = 207, 12%)	Black African (*n* = 54, 3%)	Black Other (*n* = 48, 3%)	Asian (*n* = 60, 4%)	Mixed ethnicity (*n* = 149, 9%)	Other ethnicity (*n* = 51, 3%)
1	Alcohol problem 43%	Alcohol problem 47%	Substance use 47%	Psychosis 43%	Psychosis 58%	Alcohol problem 40%	Alcohol problem 40%	Psychosis 49%
2	Substance use 43%	Substance use 46%	Psychosis 44%	Alcohol problem 37%	Bipolar disorder 40%	Psychosis 38%	Substance use 38%	Bipolar disorder 37%
3	Psychosis 37%	Psychosis 34%	Alcohol problem 40%	Depression 32%	Substance use 35%	Substance use 32%	Psychosis 37%	Alcohol problem 35%
4	Bipolar disorder 27%	Bipolar disorder 26%	Bipolar disorder 25%	Bipolar disorder 26%	Alcohol problem 27%	Bipolar disorder 30%	Bipolar disorder 28%	Substance use 29%
5	PD 21%	PD 22%	Schizophrenia 20%	Anxiety 24%	PD 25%	Depression 22%	PD 26%	Anxiety 22%

IBS, Irritable bowel syndrome; PD, Personality disorder.

### Ethnic differences in physical and mental multimorbidity status

Cross-sectional associations between ethnicity and different cut-off points for physical multimorbidity are presented in Table 3 (top half). Unadjusted and age- and gender-adjusted models are presented in online Supplementary Table S5. In fully adjusted models, rates of physical multimorbidity among working age adults of Black Caribbean ethnicity were greater for 2 (aRRR = 1.25, 95% CI 1.15–1.36) and 3 conditions (aRRR = 1.30, 95% CI 1.14–1.48) when compared to the White ethnic group. Among adults of Asian ethnicities, rates of physical multimorbidity were higher than those seen in people of White ethnicity for 3 (aRRR = 1.52, 95% CI 1.27–1.84) and 4 or more conditions (aRRR = 1.53, 95% CI 1.17–2.00). No ethnic differences emerged with regards to mental multimorbidity (bottom half of Table 3).

**Table 3.** Fully-adjusted associations between ethnicity and multimorbidity status in working-age adults with a history of common mental health disorders

	2 conditions		3 conditions		4 + conditions	
	aRRR (95% CI)	*p* value	aRRR (95% CI)	*p* value	aRRR (95% CI)	*p* value
Physical multimorbidity						
White	Reference		Reference		Reference	
Black Caribbean	**1.25 (1.15–1.36)**	**<0.001**	**1.30 (1.14–1.48)**	**<0.001**	1.10 (0.93–1.31)	0.271
Black African	0.82 (0.72–0.94)	0.006	0.87 (0.70–1.09)	0.233	0.83 (0.62–1.11)	0.216
Black Other	1.24 (1.06–1.46)	0.008	1.30 (1.02–1.65)	0.035	1.46 (1.09–1.95)	0.010
Asian	0.96 (0.84–1.10)	0.559	**1.52 (1.27–1.84)**	**<0.001**	**1.53 (1.17–2.00)**	**0.002**
Mixed ethnicity	**1.25 (1.13–1.39)**	**<0.001**	1.19 (0.99–1.43)	0.051	0.95 (0.71–1.26)	0.720
Other ethnicity	**0.78 (0.67–0.90)**	**0.001**	0.83 (0.66–1.06)	0.135	0.93 (0.69–1.25)	0.630
Mental multimorbidity						
White	Reference		Reference		Reference	
Black Caribbean	0.92 (0.77–1.10)	0.364	0.81 (0.56–1.17)	0.257	0.92 (0.51–1.67)	0.794
Black African	0.78 (0.57–1.06)	0.111	0.59 (0.29–1.21)	0.155	0.23 (0.03–1.67)	0.146
Black Other	0.86 (0.60–1.23)	0.400	1.45 (0.83–2.52)	0.191	0.30 (0.04–2.20)	0.239
Asian	0.85 (0.64–1.14)	0.287	0.66 (0.34–1.30)	0.231	–	–
Mixed ethnicity	1.24 (1.01–1.52)	0.036	1.39 (0.94–2.07)	0.102	0.93 (0.40–2.15)	0.864
Other ethnicity	0.73 (0.53–1.01)	0.057	0.76 (0.40–1.44)	0.405	0.23 (0.03–1.64)	0.141

*Covariates: age, sex, neighbourhood deprivation, BMI, smoking status, number of IAPT episodes.

Bonferroni corrected alpha level = 0.003 – significant associations are highlighted in bold.

### Ethnic differences in physical and mental multimorbidity clusters

In working age adults with physical multimorbidity, the Calinski-Harabasz *F* statistic indicated that the optimal number of clusters was three (online Supplementary Fig. S1). As Table 4 data illustrates, Cluster 1 contained the most patients (71%) and comprised psoriasis/eczema and asthma forming an atopic multimorbidity cluster. Cluster 2 (18%) contained 15 conditions that occurred at a higher-than-average prevalence. The conditions with the highest O/E ratios [diabetes, hypertension, chronic kidney disease (CKD), coronary heart disease (CHD), and peripheral vascular disease (PVD)] indicated that cluster 2 formed a cardiometabolic cluster. The third cluster (10%) contained 21 conditions and formed a mixed/multisystem multimorbidity group. In fully adjusted multinomial logistic regression analyses (Table 4), working age adults from almost all ethnic minority backgrounds were more likely to belong to the cardiometabolic cluster compared to adults from a White ethnic background. For example, working age adults with physical multimorbidity of Black Caribbean ethnicity were almost twice as likely to be in the cardiometabolic cluster compared to those of White ethnicity (aRRR = 1.89, 95% CI 1.65–2.17).

**Table 4.** Profiles of physical multimorbidity in the overall sample with physical multimorbidity and the results from a multinomial logistic regression estimating associations between ethnicity and cluster membership

	Cluster 1 (*n* = 7068, 71.2%)		Cluster 2 (*n* = 1821, 18.3%)		Cluster 3 (*n* = 1034, 10.4%)	
Cluster description	Atopic multimorbidity		Cardiovascular/metabolic		Mixed/multisystem multimorbidity	
Number of physical conditions [Median (IQR)]	2 (2–3)		3 (2–4)		2 (2–2)	
Conditions with O/E ratio >1.2	Psoriasis/eczema (1.36), asthma (1.26)		Diabetes (4.12), hypertension (3.88), CKD (3.34), CHD (3.26), PVD (3.32), heart failure (3.25), atrial fibrillation (2.94), blindness/visual impairment (2.50), glaucoma (2.05), liver disease (2.05), diverticular disease (1.63), peptic ulcer (1.51), COPD (1.36), cancer (1.35), prostate conditions (1.29)		Dementia (4.2), osteoporosis (3.2), multiple sclerosis (2.6), cancer (2.6), HIV (2.5), epilepsy (2.5), chronic sinusitis (2.4), IBD (2.4), thyroid problems (2.3), peptic ulcer (2.23), liver disease (2.2), prostate conditions (2.2), bronchiectasis (2.2), hearing problems (2.1), IBS (1.9), COPD (1.9), rheumatoid arthritis/connective tissue disorders (1.7)	
Age [Median (IQR)]	39.1 (31.8–50.7)		56.0 (49.1–60.6)		47.1 (38.6–55.8)	
Female	4820 (68.2%)		1073 (58.9%)		678 (65.6%)	
	aRRR (95% CI)	*p* value	aRRR (95% CI)	*p* value	aRRR (95% CI)	*p* value
White	Ref		Ref		Ref	
Black Caribbean	**1.23 (1.14–1.34)**	**<0.001**	**1.89 (1.65–2.17)**	**<0.001**	**0.52 (0.41–0.67)**	**<0.001**
Black African	**0.67 (0.58–0.77)**	**<0.001**	**1.78 (1.45–2.19)**	**<0.001**	**0.69 (0.49–0.97)**	**0.034**
Black Other	**1.35 (1.16–1.57)**	**<0.001**	**1.64 (1.27–2.11)**	**<0.001**	**0.53 (0.33–0.86)**	**0.010**
Asian	1.08 (0.96–1.22)	0.193	**1.75 (1.40–2.21)**	**<0.001**	0.84 (0.62–1.14)	0.266
Mixed ethnicity	**1.24 (1.12–1.36)**	**<0.001**	**1.34 (1.06–1.68)**	**0.012**	0.92 (0.71–1.20)	0.557
Other ethnicity	**0.74 (0.64–0.86)**	**<0.001**	1.15 (0.90–1.49)	0.263	0.85 (0.62–1.15)	0.290

CHD, coronary heart disease; CKD, chronic kidney disease; COPD, chronic obstructive pulmonary disorder; HIV, human immunodeficiency virus; IBD, inflammatory bowel disease; IBS, irritable bowel syndrome; PVD, peripheral vascular disease.

Covariates: Age, gender, neighbourhood deprivation, BMI, smoking status, number of IAPT episodes.

Significant associations are highlighted in bold.

O/E ratio, observed/expected ratio – the ratio of prevalence in the cluster (observed) to prevalence in the total sample (expected).

Similar to physical multimorbidity, cluster analysis indicated that the optimal number of mental multimorbidity clusters was three (online Supplementary Fig. S2). As illustrated in Table 5, Cluster 1 contained the largest proportion of working age adults with mental multimorbidity (43%) defined here as the alcohol/substance misuse multimorbidity cluster as these conditions had O/E ratios that met the cut-off for inclusion (>1.2). Cluster 2 comprised a common and severe mental health disorder multimorbidity cluster (39%), and cluster 3 comprised a personality disorder cluster (18%). Multinomial logistic regression analyses revealed that working age adults of Black African, Black Other, Asian, or Other ethnicity were less likely to belong to the alcohol/substance misuse cluster relative to adults of White ethnicity.

**Table 5.** Profiles of mental multimorbidity in the overall sample with mental multimorbidity and the results from a multinomial logistic regression estimating associations between ethnicity and cluster membership

	Cluster 1 (*n* = 731, 43%)		Cluster 2 (*n* = 651, 39%)		Cluster 3 (*n* = 300, 18%)	
Cluster description	Alcohol/substance misuse		Common and severe mental health disorders		Personality disorder	
Number of mental conditions [Median (IQR)]	2 (2–2)		2 (2–2)		2 (2–3)	
Conditions with O/E ratio >1.2	Alcohol problem (1.9), substance misuse (1.9)		Anxiety (2.0), bipolar disorder (2.0), depression (1.8), psychosis (1.7), schizophrenia (1.7)		Personality disorder (4.7), learning disability (1.5), eating disorders (1.4)	
Age [Median (IQR)]	46.4 (38.2–54.4)		39.0 (32.1–49.6)		42.5 (35.1–52.8)	
Female	298 (41%)		416 (64%)		188 (63%)	
	aRRR (95% CI)	*p* value	aRRR (95% CI)	*p* value	aRRR (95% CI)	*p* value
White	Ref		Ref		Ref	
Black Caribbean	0.89 (0.71–1.12)	0.326	1.05 (0.82–1.34)	0.714	**0.63 (0.43–0.94)**	**0.023**
Black African	**0.59 (0.36–0.95)**	**0.029**	0.98 (0.67–1.43)	0.903	**0.40 (0.18–0.92)**	**0.030**
Black Other	**0.52 (0.29–0.93)**	**0.028**	1.30 (0.85–1.98)	0.229	1.15 (0.63–2.08)	0.647
Asian	**0.64 (0.41–0.98)**	**0.043**	1.20 (0.85–1.71)	0.300	**0.21 (0.07–0.66)**	**0.007**
Mixed ethnicity	0.98 (0.73–1.32)	0.903	**1.48 (1.14–1.93)**	**0.004**	1.38 (0.95–2.01)	0.088
Other ethnicity	**0.48 (0.29–0.79)**	**0.004**	1.02 (0.68–1.53)	0.909	0.65 (0.33–1.28)	0.213

Covariates: Age, gender, neighbourhood deprivation, BMI, smoking status, number of IAPT episodes.

Significant associations are highlighted in bold.

O/E ratio, observed/expected ratio – the ratio of prevalence in the cluster (observed) to prevalence in the total sample (expected).

## Discussion

In the current study, we observed significant ethnic variation in levels of physical multimorbidity among working age adults from South London with a history of common mental health disorders who had accessed IAPT services. However, we did not observe any ethnic differences in levels of mental multimorbidity. Overall, working age adults from most ethnic minority groups showed higher rates of physical multimorbidity compared to adults of White ethnicity, with the exception of adults of Black African ethnicity where a negative association emerged (2 conditions). However, this became unsignificant after correction for multiple testing. Nevertheless, such findings underline the need to disaggregate broad ethnic groups into more specific groups. Observed ethnic differences in physical multimorbidity varied with the choice of cut-off point. For example, adults of Asian ethnicity presented with higher rates of physical multimorbidity when using higher thresholds (3 and 4 or more conditions), while this was the case among adults of Black Caribbean ethnicity with 2 and 3 conditions, but not 4 or more conditions. No significant ethnic differences emerged for mental multimorbidity. Finally, cluster analyses revealed that a 3-cluster model fitted the data best for both physical and mental multimorbidity. In general, almost all ethnic minority groups had a higher probability of belonging to the cardiometabolic physical multimorbidity cluster and were less likely to belong to the mental health multimorbidity cluster characterised by alcohol/substance misuse.

Disaggregating ethnicity data in the current study revealed notable intra-ethnic differences in physical and mental multimorbidity among working age adults. These differences may reflect the proposed heterogeneity of Black ethnic groups with regards to personal health behaviours, differential access to health care (IAPT), early life conditions, immigration history, or intra-ethnic economic niches (Keane, Tappen, Williams, & Rosselli, 2009). For instance, adults of Black Caribbean ethnicity in this study tended to be on average 3–4 years older and presented with higher rates of current smoking behaviour compared to adults of Black African ethnicity. Working age adults from a Black Caribbean ethnic background also presented with higher baseline depression and/or anxiety scores at their first IAPT episode relative to their counterparts from a Black African or Black Other ethnic group. Given the proposed association of depression with incident physical multimorbidity (de la Torre et al., 2021), the higher burden of mental health disorders at baseline may partially account for the poor physical health status of adults of Black Caribbean and Black Other ethnicity. Further, adults of Black Caribbean ethnicity showed higher rates of inflammatory disorders (e.g. psoriasis), substance misuse and psychosis, relative to their counterparts of Black African or White ethnicity. These conditions are associated with increased unemployment, social isolation, and decreased quality of life in working age populations (Yew, Kuan, Ge, Yap, & Heng, 2020).

The healthy immigrant hypothesis may also help explain the observed differences in multimorbidity between adults from a Black ethnic background (Allen et al., 2014; Borhade & Dey, 2018; Fox, Thayer, & Wadhwa, 2017). This hypothesis suggests that newly migrated immigrants have superior health compared to locals with similar socio-demographic attributes. People of Black Caribbean ethnicity immigrated to the UK largely during the 1950s and 1960s, while immigration of people of Black African ethnicity is a more recent phenomenon. Thus, the former group may have experienced extended minority status and associated discriminatory practices regarding employment opportunities or health care access (Kirmayer et al., 2007). Several studies have reported that immigrants with shorter residency (<10 years) have superior mental health to those of longer residency (Rivera, Casal, & Currais, 2016). In line with this evidence, our study findings highlight the need to consider the interaction between ethnicity and generational status to address ethnic differences in physical and mental multimorbidity. This suggestion is made cautiously as the present study lacked relevant data (e.g. generational status) to enable direct assessment of this interaction.

Our findings are largely in agreement with studies which have reported higher rates of physical multimorbidity in Black and Asian ethnic minority groups compared to White ethnic groups (Ashworth et al., 2019; Quiñones et al., 2011; Verest et al., 2019). We report no significant ethnic differences in mental multimorbidity rates across ethnic groups which partly corresponds with the heterogeneity reported by studies which have examined ethnic differences in mental-physical multimorbidity (Bobo et al., 2016; Rocca et al., 2014; St Sauver et al., 2015). We found lower levels of physical multimorbidity in working adults of Black African ethnicity, and higher levels within Black Caribbean and Black Other ethnic groups which is in line with previous evidence. For example, Mindell et al., found that people of Black African ethnicity reported lower rates of poor self-rated health compared to people of White ethnicity, whereas the opposite emerged within people of Black Caribbean ethnicity (Mindell et al., 2014). Our cluster-based findings seem to be in contrast with evidence from a recent US study (Quiñones et al., 2021) that found that older adults of Black ethnicity were more likely to belong to a complex, multisystem multimorbidity cluster. The US study used a more restrictive number of conditions (six) and diverse sampling (adults over 50 years of age, cohort *v.* clinical population), which may account for the observed differences.

### Strengths and limitations

The present study has several strengths, including a large, ethnically diverse sample derived from linked electronic health records. The linkage enabled us to access detailed information about ethnicity and allowed us to include a large number of physical and mental health conditions prevalent within a locally representative working age population. Understanding how and why multimorbidity presents earlier in the lifecourse (i.e. working age) is important given its association with poor health trajectories and lost productivity into later life (Johnson-Lawrence et al., 2017). Examining multimorbidity in the context of common mental disorders enabled us to explore ethnic differences in a unique cohort at higher risk of poorer health trajectories and premature mortality.

Several limitations also need consideration when interpreting the study findings. While the study controlled for a number of relevant covariates, we cannot reject the possibility of unmeasured confounders (e.g. adverse life events, therapeutic interventions, generational status) that may have biased the observed association between ethnicity and multimorbidity. While our study identified several covariates as confounders, cross-sectional analyses prevented more complex modelling of these covariates (e.g. the mediating role of smoking or BMI) that would require longitudinal data. Moreover, the cross-sectional nature of the study-design did not allow insights into ethnic differences in the risk of developing physical and mental multimorbidity over time. Future prospective studies are needed to link the evolving and fluctuating nature of depression and anxiety, with ethnic differences in the trajectory of physical and mental multimorbidity across the lifespan. Missing data on ethnicity and the diversity of adults included in the Other ethnic group are important limitations of the present analyses. Moreover, sample size meant that we were unable to disaggregate Asian groups meaning that we could not to assess differences in multimorbidity between Asian ethnicities. This should be a priority for future research. The wider CIs seen when looking at ethnic differences in higher thresholds of multimorbidity (i.e. 4 or more conditions) imply small sample size and interpretations relating to this more complex multimorbidity are made with caution. While ethnically diverse, our study sample included working age adults who had previously accessed IAPT services in an urban, relatively deprived borough of London, that may restrict the generalisability of the findings to similar treatment-seeking populations with a history of depression/anxiety from different UK geographical areas. Moreover, the exclusion of people with missing data for ethnicity also poses a limitation for the interpretation of results as this led to a healthier cohort (i.e. lower levels of multimorbidity) implying that our study estimates (effect sizes) are on the conservative side. Further, due to insufficient study power, we were unable to explore interactions between ethnicity and gender. Even so, our study findings are supportive of calls to further disaggregate ethnicity data in order to better understand ethnic differences in health. We used cross-sectional primary care data to provide a snapshot of multimorbidity rates among different ethnic groups in South London. Although this was likely to provide us with a fairly comprehensive measure of multimorbidity, it is possible that it might be underestimated. The available length of medical history, which would certainly fluctuate amongst immigrants depending on time spent in the UK, might differ across ethnic groups and we were unable to account for immigrant status or length of time in contact with primary care services.

## Conclusions

Working age adults from ethnic minority backgrounds presented with substantial variation in rates of physical, but not mental, multimorbidity. The observed heterogeneity in physical multimorbidity within adults of Black Caribbean, Black Other, and Black African ethnicity validates the importance to further disaggregate broader ethnic groups to increase our understanding of ethnic differences in health and multimorbidity. Practitioners might benefit from the insight that people of Black Caribbean ethnicity have higher risk of physical multimorbidity relative to those of Black African ethnicity. Moreover, these findings have the potential to inform government policies on health inequalities, but this would benefit from more accurate ethnicity data than available in the current study. Finally, future prospective studies are needed to evaluate the predictive utility of the multimorbidity patterns we identified for clinical and functional outcomes.

## Supplementary material

For supplementary material accompanying this paper visit https://doi.org/10.1017/S0033291722003488. click here to view supplementary material
