# Supplementary material for: Ethnic differences in physical and mental multimorbidity in working age adults with a history of depression and/or anxiety
Source: Psychol Med. 2022 Nov 24;53(13):6212–22. doi: 10.1017/S0033291722003488 (PMC10520586; doi:10.1017/S0033291722003488)
Supplement: Supplementary file 1 [file S0033291722003488sup001.docx]

| Table S1. Derived ethnicity variable from IAPT data | |  |
| --- | --- | --- |
| Ethnicity | Ethnic group | Ethnic subgroup |
| White | White | White British, White Irish, White Other |
| Black Caribbean | Black/Black British | Black Caribbean |
| Black African | Black/Black British | Other African, Somali, Nigerian, Sudanese, Angolan, Eritrean, Ethiopian, Ghanaian, Ugandan |
| Black Other | Black/Black British | Mixed Black, Other Black British, Other Black or Black unspecified |
| Asian | Asian or Asian British | Indian, Pakistani, Bangladeshi, Mixed Asian, Punjabi, East African Asian, Sri Lankan, Tamil, Sinhalese, British Asian, Caribbean Asian, Other Asian or Asian unspecified, Vietnamese, Japanese, Filipino, Malaysian |
| Mixed ethnicity | Mixed | White and Black Caribbean, White and Black African, White and Asian, Black and Asian, Black and Chinese, Black and White, Chinese and White, Asian and Chinese, Other mixed or mixed unspecified |
| Other ethnic group | Other ethnic group | Chinese, Arab/Middle Eastern (Algerian, Middle Eastern, Arab, Iranian, Iraqi), Any Other Group, Columbian, Ecuadorian, Other Latin American |

| Table S2. Mental and physical conditions from linked primary care records (Lambeth DataNet) | |
| --- | --- |
| Mental and behavioural conditions |  |
| Depression | Recorded in previous 12 months |
| Anxiety | Recorded in previous 12 months |
| Bipolar disorder | Ever recorded |
| Psychosis | Ever recorded |
| Schizophrenia | Ever recorded |
| Personality disorder | Ever recorded |
| Anorexia/bulimia | Ever recorded |
| Alcohol problem | Ever recorded |
| Substance misuse | Ever recorded |
| Learning disability | Ever recorded |
| Physical long-term conditions |  |
| Cancer | Recorded in the previous 5 years |
| Human immunodeficiency virus (HIV) | Ever recorded |
| Diabetes | Ever recorded |
| Thyroid problems | Ever recorded |
| Epilepsy | Ever recorded |
| Dementia | Ever recorded |
| Multiple sclerosis (MS) | Ever recorded |
| Parkinson’s disease | Ever recorded |
| Blindness/visual impairment | Ever recorded |
| Glaucoma | Ever recorded |
| Hearing problems | Ever recorded |
| Atrial fibrillation | Ever recorded |
| Coronary heart disease (CHD) | Ever recorded |
| Heart failure | Ever recorded |
| Hypertension | Ever recorded |
| Peripheral vascular disease (PVD) | Ever recorded |
| Stroke | Ever recorded |
| Asthma | Ever recorded |
| Bronchiectasis | Ever recorded |
| Chronic obstructive pulmonary disorder (COPD) | Ever recorded |
| Chronic sinusitis | Ever recorded |
| Diverticular disease | Ever recorded |
| Irritable bowel syndrome (IBS) | Ever recorded |
| Inflammatory bowel disorder (IBD) | Ever recorded |
| Liver disease | Ever recorded |
| Peptic ulcer | Ever recorded |
| Psoriasis/eczema | Ever recorded |
| Osteoporosis | Ever recorded |
| Rheumatoid arthritis/connective tissue disorders | Ever recorded |
| Chronic kidney disease (CKD) | Ever recorded |
| Prostate conditions | Ever recorded |

| **Table S3.** Differences between the analytical sample and excluded participants on all study variables | | | |
| --- | --- | --- | --- |
|  | Analytical sample  (n=44,506) | Excluded participants  (n=2591) | P value |
| Age *(Median (IQR))* | 37.4 (31.3 to 46.6) | 61.2 (40.6 to 67.1) | <0.001 |
| Female | 29,054(65) | 1642 (63) | 0.069 |
| IMD *(Median (IQR))* | 3 (2 to 5) | 3 (2 to 5) | <0.001 |
| BMI |  |  | <0.001 |
| *Obese* | 6,788 (15) | 647 (25) |  |
| *Overweight* | 12,315(28) | 857 (33) |  |
| *Optimal* | 23,846(54) | 1027 (40) |  |
| *Underweight* | 1557 (4) | 60 (2) |  |
| Smoking status |  |  | 0.001 |
| *Current* | 14,406(32) | 801 (31) |  |
| *Ex-smoker* | 7947 (18) | 539 (21) |  |
| *Never smoked* | 22,153(50) | 1251 (48) |  |
| IAPT episodes *(Median (IQR)))* | 1 (1 to 1) | 1 (1 To 1) | 0.805 |
| PHQ-9*  *(Median (IQR))* | 15 (10 to 19) | 16 (11 to 21) | <0.001 |
| GAD-7*  *(Median (IQR))* | 14 (10 to 17) | 14 (10 to 18) | 0.003 |
| *Physical MM* | 9923 (22) | 1101 (43) | <0.001 |
| 0/1 condition | 34,583(78) | 1490 (57) |  |
| 2 conditions | 6592 (15) | 480 (19) |  |
| 3 conditions | 2162 (5) | 284 (11) |  |
| 4+ conditions | 1169 (3) | 337 (13) |  |
| *Mental MM* | 1682 (4) | 123 (5) | 0.020 |
| 0/1 condition | 42,824(96) | 2468 (95) |  |
| 2 conditions | 1293 (3) | 87 (3.4) |  |
| 3 conditions | 300 (1) | 26 (1) |  |
| 4+ conditions | 89 (0) | 10 (0.4) |  |
| BMI=Body mass index; GAD-7=Generalised anxiety disorder assessment; IAPT=Improving Access to Psychological Therapies; IMD=Index of Multiple Deprivation; PHQ-9=Patient Health Questionnaire-9  *Baseline PHQ-9 and GAD-7 scores are taken from the first episode of care in IAPT services | | | |

| **Table S4.** Fully adjusted associations between ethnicity and multimorbidity (yes/no) in working-age adults with a history of common mental health disorders – complete case analysis (N=38,292) | | | | | | |
| --- | --- | --- | --- | --- | --- | --- |
|  | **2 conditions** | | **3 conditions** | | **4+ conditions** | |
| **Unadjusted** | *RRR (95%CI)* | *p value* | *RRR (95%CI)* | *p value* | *RRR (95%CI)* | *p value* |
| **Physical multimorbidity** | | | | | | |
| White | Reference |  | Reference |  | Reference |  |
| Black Caribbean | **1.29 (1.18 to 1.41)** | **<0.001** | **1.30 (1.13 to 1.49)** | **<0.001** | 1.13 (0.95 to 1.36) | 0.170 |
| Black African | 0.83 (0.71 to 0.96) | 0.012 | 0.92 (0.73 to 1.15) | 0.476 | 0.91 (0.68 to 1.23) | 0.546 |
| Black Other | 1.21 (1.02 to 1.44) | 0.027 | 1.34 (1.04 to 1.73) | 0.023 | 1.50 (1.11 to 2.03) | 0.008 |
| Asian | 0.95 (0.83 to 1.10) | 0.512 | **1.54 (1.27 to 1.87)** | **<0.001** | **1.65 (1.26 to 2.17)** | **<0.001** |
| Mixed ethnicity | **1.25 (1.12 to 1.39)** | **<0.001** | 1.23 (1.02 to 1.48) | 0.030 | 0.98 (0.73 to 1.32) | 0.910 |
| Other ethnicity | **0.78 (0.67 to 0.92)** | **0.003** | 0.93 (0.73 to 1.18) | 0.541 | 0.92 (0.67 to 1.27) | 0.624 |
| **Mental multimorbidity** | | | | | | |
| White | Reference |  | Reference |  | Reference |  |
| Black Caribbean | 0.95 (0.70 to 1.15) | 0.634 | 0.79 (0.54 to 1.16) | 0.225 | 1.03 (0.57 to 1.88) | 0.912 |
| Black African | 0.80 (0.58 to 1.11) | 0.189 | 0.55 (0.26 to 1.18) | 0.125 | 0.26 (0.03 to 1.88) | 0.181 |
| Black Other | 0.89 (0.61 to 1.29) | 0.538 | 1.42 (0.80 to 2.53) | 0.228 | 0.35 (0.04 to 1.88) | 0.294 |
| Asian | 0.83 (0.61 to 1.12) | 0.225 | 0.54 (0.25 to 1.15) | 0.110 | - |  |
| Mixed ethnicity | 1.28 (1.04 to 1.58) | 0.021 | 1.36 (0.90 to 2.05) | 0.142 | 0.85 (0.34 to 2.14) | 0.735 |
| Other ethnicity | 0.73 (0.52 to 1.03) | 0.075 | 0.666 (0.32 to 1.34) | 0.249 | 0.25 (0.03 to 1.84) | 0.176 |
| *Covariates: age, sex, neighbourhood deprivation, BMI, smoking status, number of IAPT episodes  Bonferroni corrected alpha level = 0.003 – significant associations are highlighted in bold | | | | | | |

| **Table S5.** Unadjusted and age- and gender-adjusted associations between ethnicity and multimorbidity (yes/no) in working-age adults with a history of common mental health disorders | | | | | | |
| --- | --- | --- | --- | --- | --- | --- |
|  | **2 conditions** | | **3 conditions** | | **4+ conditions** | |
| **Unadjusted** | *RRR (95%CI)* | *p value* | *RRR (95%CI)* | *p value* | *RRR (95%CI)* | *p value* |
| **Physical multimorbidity** | | | | | | |
| White | Reference |  | Reference |  | Reference |  |
| Black Caribbean | 1.53 (1.41 to 1.66) | <0.001 | 2.00 (1.76 to 2.26) | <0.001 | 2.24 (1.91 to 2.63) | <0.001 |
| Black African | 0.93 (0.82 to 1.07) | 0.331 | 1.16 (0.93 to 1.43) | 0.180 | 1.33 (1.02 to 1.74) | 0.038 |
| Black Other | 1.54 (1.32 to 1.81) | <0.001 | 2.06 (1.63 to 2.60) | <0.001 | 3.04 (2.33 to 3.97) | <0.001 |
| Asian | 1.01 (0.88 to 1.14) | 0.935 | 1.65 (1.38 to 1.98) | <0.001 | 1.58 (1.23 to 2.02) | <0.001 |
| Mixed ethnicity | 1.25 (1.13 to 1.38) | <0.001 | 1.16 (0.98 to 1.38) | 0.090 | 0.88 (0.67 to 1.14) | 0.337 |
| Other ethnicity | 0.88 (0.76 to 1.02) | 0.094 | 1.09 (0.86 to 1.37) | 0.480 | 1.38 (1.04 to 1.83) | 0.027 |
| **Mental multimorbidity** | | | | | | |
| White | Reference |  | Reference |  | Reference |  |
| Black Caribbean | 1.14 (0.96 to 1.36) | 0.125 | 1.11 (0.77 to 1.59) | 0.578 | 1.30 (0.73 to 2.33) | 0.360 |
| Black African | 0.77 (0.57 to 1.04) | 0.088 | 0.60 (0.29 to 1.21) | 0.153 | 0.22 (0.03 to 1.59) | 0.133 |
| Black Other | 1.04 (0.73 to 1.48) | 0.833 | 1.92 (1.11 to 3.32) | 0.019 | 0.41 (0.06 to 2.93) | 0.372 |
| Asian | 0.83 (0.62 to 1.10) | 0.199 | 0.64 (0.33 to 1.25) | 0.191 | - | - |
| Mixed ethnicity | 1.26 (1.03 to 1.54) | 0.022 | 1.40 (0.95 to 2.08) | 0.090 | 0.86 (0.37 to 1.98) | 0.719 |
| Other ethnicity | 0.80 (0.58 to 1.10) | 0.165 | 0.87 (0.46 to 1.64) | 0.669 | 0.26 (0.04 to 1.85) | 0.178 |
| **Age- and gender-adjusted** | *RRR (95%CI)* | *p value* | *RRR (95%CI)* | *p value* | *RRR (95%CI)* | *p value* |
| **Physical multimorbidity** | | | | | | |
| White | Reference |  | Reference |  | Reference |  |
| Black Caribbean | 1.39 (1.29 to 1.51) | <0.001 | 1.58 (1.40 to 1.80) | <0.001 | 1.44 (1.22 to 1.71) | <0.001 |
| Black African | 0.93 (0.81 to 1.06) | 0.277 | 1.10 (0.89 to 1.36) | 0.395 | 1.15 (0.86 to 1.52) | 0.341 |
| Black Other | 1.39 (1.19 to 1.63) | <0.001 | 1.62 (1.27 to 2.06) | <0.001 | 1.98 (1.49 to 2.63) | <0.001 |
| Asian | 0.99 (0.87 to 1.13) | 0.918 | 1.61 (1.34 to 1.93) | <0.001 | 1.54 (1.18 to 2.00) | 0.001 |
| Mixed ethnicity | 1.32 (1.20 to 1.46) | <0.001 | 1.33 (1.11 to 1.58) | 0.002 | 1.10 (0.83 to 1.46) | 0.482 |
| Other ethnicity | 0.82 (0.71 to 0.95) | 0.008 | 0.91 (0.72 to 1.16) | 0.465 | 1.03 (0.77 to 1.39) | 0.836 |
| **Mental multimorbidity** | | | | | | |
| White | Reference |  | Reference |  | Reference |  |
| Black Caribbean | 1.06 (0.89 to 1.26) | 0.492 | 0.96 (0.67 to 1.38) | 0.823 | 1.05 (0.59 to 1.89) | 0.858 |
| Black African | 0.77 (0.57 to 1.05) | 0.101 | 0.59 (0.29 to 1.20) | 0.144 | 0.21 (0.03 to 1.53) | 0.125 |
| Black Other | 0.95 (0.67 to 1.36) | 0.790 | 1.64 (0.95 to 2.84) | 0.078 | 0.32 (0.04 to 2.32) | 0.261 |
| Asian | 0.82 (0.61 to 1.09) | 0.165 | 0.62 (0.32 to 1.22) | 0.166 | - | - |
| Mixed ethnicity | 1.39 (1.14 to 1.70) | 0.001 | 1.59 (1.07 to 2.36) | 0.021 | 1.03 (0.46 to 2.39) | 0.935 |
| Other ethnicity | 0.75 (0.54 to 1.04) | 0.080 | 0.79 (0.41 to 1.49) | 0.463 | 0.22 (0.03 to 1.61) | 0.137 |
|  | | | | | | |

Figure S1. Calinski-Harabasz indices for physical multimorbidity clusters. Results indicate a three-cluster model is optimal.

0

Figure S2. Calinski-Harabasz indices for mental multimorbidity clusters. Results indicate a three-cluster model is optimal.
